# MICAL-L2 Is Essential for c-Myc Deubiquitination and Stability in Non-small Cell Lung Cancer Cells

**DOI:** 10.3389/fcell.2020.575903

**Published:** 2021-01-14

**Authors:** Pengxiang Min, Lin Zhang, Yueyuan Wang, Chenxiang Qi, Yixuan Song, Maria Bibi, Yujie Zhang, Yadong Ma, Xuyang Zhao, Minjie Yu, Jun Du

**Affiliations:** ^1^Department of Physiology, Nanjing Medical University, Nanjing, China; ^2^Key Laboratory of Cardiovascular & Cerebrovascular Medicine, School of Pharmacy, Nanjing Medical University, Nanjing, China; ^3^Department of Pathology, Xuzhou Medical University, Xuzhou, China; ^4^Jiangsu Key Lab of Cancer Biomarkers, Prevention and Treatment, Collaborative Innovation Center for Cancer Personalized Medicine, Nanjing Medical University, Nanjing, China; ^5^Department of Biochemistry and Molecular Biology, Nanjing Medical University, Nanjing, China; ^6^The First Clinical Medical College, Nanjing Medical University, Nanjing, China

**Keywords:** c-Myc, MICAL-L2, NSCLC, proliferation, deubiquitination

## Abstract

**Objectives:** MICAL-L2, a member of the molecules interacting with the CasL (MICAL) family, was reported to be highly expressed in several types of cancers, however, the roles of MICAL-L2 in NSCLC pathogenesis remain to be explored. This study is designed to clarify the mechanisms by which MICAL-L2 participates in NSCLC cell proliferation.

**Materials and Methods:** The expression levels of MICAL-L2 in human lung cancer samples were assessed by immunohistochemical staining. Cells were transfected with siRNA or plasmids to regulate MICAL-L2 expression. Cell proliferation was measured by EdU staining and CCK-8 assays. MICAL-L2 and phosphorylated/total c-Myc expression were examined by Western blotting analysis. Interaction between MICAL-L2 and c-Myc was assessed by immunofluorescence staining, Western blotting and co-immunoprecipitation assays. Western blotting, polyubiquitylation detection and protein stability assays were used to assess whether MICAL-L2 exerts its oncogenic effect via c-Myc.

**Results:** We found that MICAL-L2 was highly expressed in human NSCLC. While overexpressing MICAL-L2 increased NSCLC cell proliferation, MICAL-L2 depletion decreased the proliferation of NSCLC cells, an effect that was linked to cell cycle arrest. MICAL-L2 physically interacted with the c-Myc protein and functioned to maintain nuclear c-Myc levels and prolonged its half-life. Knockdown of MICAL-L2 expression led to decreased c-Myc protein stability through accelerating polyubiquitylation of c-Myc and gave rise to c-Myc degradation. We further found that MICAL-L2 deubiquitinated c-Myc and blocked its degradation, presumably by inhibiting c-Myc phosphorylation at threonine residue 58.

**Conclusions:** These results indicate that MICAL-L2 is a key regulator of c-Myc deubiquitination and stability in the nucleus, and this activity may be involved in promoting NSCLC cell proliferation.

## Introduction

Lung cancer is the most common type of malignant tumor and one of the malignancies with the fastest increase in morbidity and mortality worldwide (Torre et al., 2016). Non-small cell lung cancer (NSCLC) accounts for more than 80% of all lung cancer cases. Approximately 75% of NSCLC patients are diagnosed in the middle and advanced stages of the disease, and the 5-year survival rate is very low. c-Myc, an oncogene-encoded protein, is highly expressed in NSCLC and plays a key role in its carcinogenesis (Wu et al., 2015; Li et al., 2019). Consequently, understanding the mechanisms that control the level and activity of c-Myc in NSCLC is important to support the development of novel therapeutic interventions.

c-Myc is a multifunctional transcription factor with roles in various cellular processes, including metabolism and growth (Dang, 2012). Normally, dysregulated c-Myc expression, rather than the expression of a mutated form of the protein, is responsible for its oncogenic effects. Its expression could be influenced at the transcriptional level by c-Myc gene amplification. Protein stability is also an important mechanism underlying the regulation of c-Myc protein content owing to its short half-life in proliferating cells. c-Myc has been identified to undergo phosphorylation modification by the proteasome, which triggers c-Myc ubiquitylation and degradation (Hann, 2006; Farrell and Sears, 2014). Meanwhile, the ubiquitination of c-Myc can be reversed by deubiquitinating enzymes such as USP28 and USP36 (Popov et al., 2007; Sun et al., 2015). The degradation of c-Myc has recently also been found to be regulated through a ubiquitin-independent pathway, i.e., autophagic lysosomal degradation (Liu et al., 2018; Murai et al., 2018). Generally, c-Myc is regulated and exerts its oncogenic activity in the nucleus. However, whether c-Myc is deubiquitinated in the nucleus in NSCLC cells remains to be determined.

Remodeling of the actin cytoskeleton, a molecular framework that provides physical support for cell structure and proliferation, depends on the spatial regulation of an upstream signaling network (Giridharan and Caplan, 2014; Roy and Burkhardt, 2018; Hohmann and Dehghani, 2019; Phuyal and Farhan, 2019). Molecules interacting with CasL (MICALs) comprise a family of flavoprotein monooxygenases that interact with F-actin and participate in a multitude of activities related to cytoskeleton dynamics. MICAL-L2, a member of the MICAL family, is a recently identified Rab13-binding protein and coordinates with Rab13 to promote neurite outgrowth (Rahajeng et al., 2010; Sakane et al., 2010). MICAL-L2 has been found to be highly expressed in multiple types of cancer, including ovarian, gastric, and breast cancers (Ioannou et al., 2015; Zhu et al., 2015; Min et al., 2019). Recent studies have shown that MICAL-L2 localization to the leading edge of migrating cells is necessary for the metastatic behavior of cancer cells (Ioannou et al., 2015; Sakane et al., 2018). Meanwhile, the silencing of MICAL-L2 was reported to inhibit both the nuclear translocation of beta-catenin and ovarian cancer cell proliferation (Zhu et al., 2015). We have also recently shown that MICAL-L2 attenuates lysosome-mediated EGFR degradation and enhances the migratory ability of gastric cancer cells, suggesting that MICAL-L2 functions to prevent the degradation of its client proteins and may play an oncogenic role in gastric cancer (Min et al., 2019). Although MICAL-1, another member of the MICAL family, was reported to exert its effect on breast cancer cell proliferation via ROS-sensitive PI3K/AKT/ERK signaling (Deng et al., 2018), the function of MICAL-L2 in NSCLC cell proliferation and progression is largely unknown.

Interestingly, proteomic profiling has demonstrated that MICAL-L2 interacts with c-Myc (Agrawal et al., 2010). Strong MICAL-L2 staining has also been observed in the nuclei of ovarian cancer tissues (Zhu et al., 2015). Consequently, we hypothesized that MICAL-L2 can promote NSCLC cell proliferation through binding to c-Myc and may help attenuate c-Myc degradation. In the present study, we found that MICAL-L2 was highly expressed in NSCLC tissues and cells, and its expression was associated with cell proliferation. We further found that MICAL-L2 acts as a positive regulator of c-Myc protein stabilization by preventing its ubiquitin-dependent degradation in the nucleus. These findings indicate that MICAL-L2 promotes NSCLC cell proliferation by maintaining c-Myc content.

## Materials and Methods

### Ethics Statement

All immunohistochemistry assays with human tumor specimens were conducted under the institutional guidelines of Jiangsu Province.

### Cell Culture

The human NSCLC cell lines A549, NCI-H1299, NCI-H292, and PC9; the normal human fibroblast cell line MRC5; and the human bronchial epithelial cell line BEAS-2B were obtained from the Cell Biology Institute of the Chinese Academy of Science (Shanghai, China). All the cells were maintained in Dulbecco's modified Eagle's medium (DMEM, high glucose) or RPMI 1640 (Hyclone, Thermo Scientific, Waltham, MA, USA) supplemented with 10% fetal bovine serum (FBS; Gibco, Carlsbad, CA, USA), penicillin (100 U/mL), and streptomycin (100 μg/mL) at 37°C in a humidified incubator with 5% CO_2_. Cells were grown on plastic dishes for RNA isolation and protein extraction and coverslips for fluorescence staining.

### Plasmids and siRNAs

Full-length *MICALL2* was PCR-amplified from the pCMV-SPORT6-MICAL-L2 plasmid (YouBio, Hunan, China) and cloned into the pCMV-C-HA or pEGFP-N1 vector (Beyotime, Nantong, China) as previously described (Min et al., 2019). For plasmid construction, the cDNA of the c-Myc gene was amplified by PCR from NCI-H1299 cells and inserted into the pCMV-N-Flag vector (Beyotime). All the constructs were verified by sequencing. When the cells had reached ~80% confluence, they were transfected with the relevant plasmids using Lipofectamine 2000 (Invitrogen, Carlsbad, CA, USA) according to the manufacturer's instructions.

siRNAs targeting MICAL-L2 were purchased from China GenePharma Co., and contained the following sequences: siMICAL-L2 #1, 5′-GGUUCCCACAAAGAGUAUATT-3′; siMICAL-L2 #2, 5′-CUCGACGUUUGUGACAACUTT-3′; siMICAL-L2 #3, 5′-CCAAGUUCCGCUUGUCCAATT-3′. The cells were transfected with MICAL-L2 siRNA or control siRNA using Lipofectamine 2000 at 80% confluence.

The transfected cells were treated with cycloheximide (CHX) (Sigma-Aldrich, Saint Louis, MO, USA), MG-132 (Selleck Chemicals, Houston, TX. USA), Velcade (Selleck Chemicals), acadesine (AICAR; Selleck Chemicals), or chloroquine diphosphate (Chlq; MedChemExpress, Monmouth, Junction, NJ, USA) at the indicated time points.

### Cell Counting Kit-8 Assay

Cell viability was detected by Cell Counting Kit-8 (CCK-8) assay. Briefly, cells were seeded in a 96-well plate and then transfected with siRNA or plasmids. After culturing for the indicated times, the culture medium was adjusted to 90 μL per well, and 10 μL of the CCK-8 solution (Selleck Chemicals) was added to each well for 1 h. The OD of each sample was measured at 450 nm using a microplate reader (Bio-Tek, Elx800, VT. USA). Each group had five replicates.

### Ethynyl-2-deoxyuridine (EdU) Incorporation Assays

Cell proliferation was further measured using an EdU staining kit (Ribobio). In brief, cells were seeded into a 96-well plate and 0.2 μL of EdU was added to the medium for 2 h. The cells were then washed with PBS, fixed in formaldehyde for 30 min, incubated with glycine, and washed with PBS containing 0.5% Triton X-100. The cells were stained with Apollo and then counterstained with Hoechst 33342. The number of EdU-labeled cells was determined using a fluorescence microscope (Carl Zeiss Meditec, Jena, Germany) and normalized to the total number of Hoechst 33342-stained cells.

### Flow Cytometry

Cell cycle analysis was conducted by flow cytometry. Briefly, at 48 h post-transfection, cells were harvested and suspended in 75% ice-cold ethanol at a concentration of 1.5 × 10^6^ cells/mL. RNase and propidium iodide staining solution were then added to the cell suspension and the mixture was incubated for 30 min at 37°C in the dark. The cell cycle distribution of the stained cells was analyzed by flow cytometry.

### Western Blotting

Cells were homogenized in lysis buffer (Beyotime) and protein concentrations were measured using a BCA Protein Assay Kit (Thermo Fisher Scientific). Equal amounts of total protein were separated by SDS-PAGE and transferred to pure nitrocellulose membranes. The bands were visualized using enhanced chemiluminescence (Millipore, Billerica, MA, USA). Digital images of the positive bands were analyzed with Quantity One software (Bio-Rad, Hercules, CA, USA). Antibodies targeting the following proteins were used: GAPDH (G9545, Sigma-Aldrich), MICAL-L2 (PA5-24826, Thermo Fisher Scientific), c-Myc (#13877, Cell Signaling Technology, Danvers, MA, USA), cyclin-D1 (#2978, Cell Signaling Technology), ubiquitin (#3936, Cell Signaling Technology), GFP (#2956, Cell Signaling Technology), CDK2 (sc-6248, Santa Cruz Biotechnology, Santa Cruz, CA, USA), CDK4 (sc-260, Santa Cruz Biotechnology), CDK6 (sc-7961, Santa Cruz Biotechnology), phosphorylated-c-Myc T58 (AF3055, Affinity, Cincinnati, OH, USA), HA (51064-2-AP, Proteintech, Wuhan, China), and Flag (M20008, Abmart, Shanghai, China).

### Real-Time Quantitative PCR

Total RNA was extracted using Trizol reagent (Invitrogen) and reverse transcription was performed using HiScript®Q RT SuperMix (Vazyme, Nanjing, China) according to the manufacturer's instructions. qPCR was performed using AceQ® qPCR SYBR® Green Master Mix (High ROX Premixed) (Vazyme) in an ABI StepOne™ Real-Time PCR System (Applied Biosystems, Foster City, CA, USA). mRNA expression levels were quantified using the 2^Δ*ΔCT*^ method (Applied Biosystems). The sequences of the primers used are listed in Table 1.

**Table 1:** Primer sequences used for qRT-PCR.

**Gene**	**Sequence**
GAPDH	5′-CATCAGCAATGCCTCCTGCAC-3′ 5′-TGAGTCCTTCCACGATACCAAAGTT-3′
MICAL-L2	5′-TGTGGTCCAGAGGAGGAATGA-3′ 5′-CAGCTCCGGTGGTAAAGCC-3′
c-Myc	5′-GTCAAGAGGCGAACACACAAC-3′ 5′-TTGGACGGACAGGATGTATGC-3′
CCNA	5′-GGATGGTAGTTTTGAGTCACCAC-3′ 5′-CACGAGGATAGCTCTCATACTGT-3′
CCNB	5′-TTGGGGACATTGGTAACAAAGTC-3′ 5′-ATAGGCTCAGGCGAAAGTTTTT-3′
CCNC	5′-CCTTGCATGGAGGATAGTGAATG-3′ 5′-AAGGAGGATACAGTAGGCAAAGA-3′
CCND1	5′-CAATGACCCCGCACGATTTC-3′ 5′-CATGGAGGGCGGATTGGAA-3′
CCND2	5′-ACCTTCCGCAGTGCTCCTA-3′ 5′-CCCAGCCAAGAAACGGTCC-3′
CCND3	5′-TACCCGCCATCCATGATCG-3′ 5′-AGGCAGTCCACTTCAGTGC-3′
CCNE	5′-ACTCAACGTGCAAGCCTCG-3′ 5′-GCTCAAGAAAGTGCTGATCCC-3′
CCNF	5′-GGAAAGCGACAGGAGGACAG-3′ 5′-TGGCAGACGATCTCACTGGAA-3′
CCNG1	5′-GAGTCTGCACACGATAATGGC-3′ 5′-GTGCTTGGGCTGTACCTTCA-3′
CCNG2	5′-TCTCGGGTTGTTGAACGTCTA-3′ 5′-GTAGCCTCAATCAAACTCAGCC-3′
CCNH	5′-AGGCACTTGAACAGATACTGGA-3′ 5′-CCAATATGGGATAGCGGGTCT-3′
CDK2	5′-CCAGGAGTTACTTCTATGCCTGA-3′ 5′-TTCATCCAGGGGAGGTACAAC-3′
CDK4	5′-GGGGACCTAGAGCAACTTACT-3′ 5′-CAGCGCAGTCCTTCCAAAT-3′
CDK6	5′-TCTTCATTCACACCGAGTAGTGC-3′ 5′-TGAGGTTAGAGCCATCTGGAAA-3′

### Protein Stability Assays

In brief, cells were incubated in the presence of cycloheximide (CHX) for 0, 30, 60, 90, 120 min. Then, the cells were collected and lysed, and the lysates were separated by SDS-PAGE for western blotting analysis of protein abundance at each time point.

### Immunofluorescence

The cells used for immunostaining were fixed in 4% paraformaldehyde for 15 min, permeabilized in 0.2% Triton X-100, and blocked with 1% BSA for 1 h at room temperature. The cells were then incubated with antibodies against MICAL-L2 (sc-376675, Santa Cruz Biotechnology) and c-Myc (sc-764x; Santa Cruz Biotechnology) at 4°C overnight followed by incubation with species-matched Alexa- or TRITC-conjugated secondary antibodies for 1 h at room temperature in a moist chamber. Cell nuclei were counterstained with DAPI (Southern Biotech, Birmingham, AL, USA). Images were acquired using an Olympus BX43 microscope (Olympus, Tokyo, Japan) coupled to an Olympus DP74 digital camera.

### Co-immunoprecipitation (Co-IP) Assay

Co-IP was conducted as previously described (Deng et al., 2016). In brief, cellular proteins were prepared and incubated with antibody at 4°C overnight. Antibody-protein complexes were precipitated using protein A + G agarose beads (Beyotime). The agarose-associated antibody-protein complexes were then dissolved in SDS-PAGE and the bound proteins were probed with antibodies as indicated in the figure legends.

### Polyubiquitination Assays

Polyubiquitination assays were conducted using Co-IP. Briefly, cells were transfected with HA-ubiquitin and the relevant plasmids. Cell lysates were harvested at 48 h post-transfection; 80% of each cell lysate was used for Co-IP and the rest was subjected to Western blotting under denaturing conditions. The bound proteins were analyzed using Western blotting.

### Immunohistochemistry

Lung adenocarcinoma (LUAD) tissue microarrays were purchased from Outdo Biotech (Shanghai, China). Thirty LUAD case samples and their corresponding paracancerous tissues were used in this study. Briefly, the microarray tissues were deparaffinized, rehydrated, blocked with 3% BSA, and incubated with antibodies against MICAL-L2 (PA5-24826, Thermo Fisher Scientific) and c-Myc (bs4963R, Biosynthesis Biotechnology Co., Ltd). After 1 h of incubation with biotinylated secondary antibodies and subsequent DAB solution staining, the samples were observed under a microscope, counterstained with hematoxylin, and then coverslipped with neutral gum. MICAL-L2 and c-Myc immunoreactivity was semi-quantified using the immunoreactive score (IRS).

### Statistical Analysis

All data were expressed as means ± S.E.M. Statistical analysis was performed using SPSS version 19.0 (SPSS, Chicago, IL, USA). The Student's *t*-test was used for comparisons between two groups.

## Results

### The Expression and Relationship of MICAL-L2 and c-Myc in Human LUAD Samples

**Figure 1:**
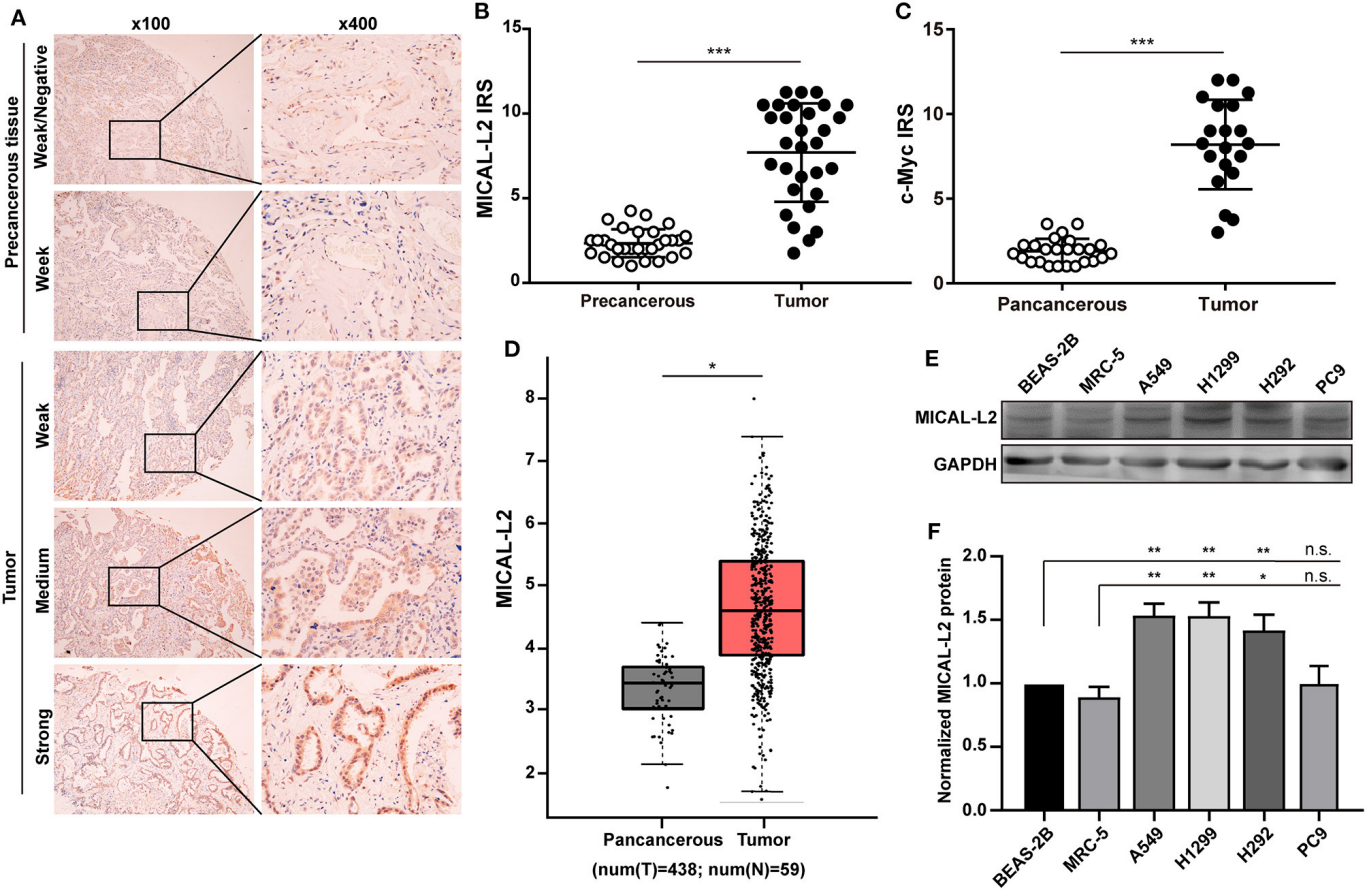
Analysis of MICAL-L2 and c-Myc expression in lung adenocarcinoma (LUAD) tissues. **(A)** Immunohistochemical staining for MICAL-L2 in LUAD tissues. **(B,C)** Analysis of MICAL-L2 and c-Myc staining in LUAD tissues. **(D)** Analysis of The Cancer Genome Atlas (TCGA) database showed that MICAL-L2 is highly expressed in lung cancer tissues when compared with normal tissues. **(E,F)** MICAL-L2 protein expression in different non-small cell lung carcinoma (NSCLC) cell lines. *P < 0.05, **P < 0.01, ***P < 0.001.

The expression of MICAL-L2 and c-Myc was evaluated in a tissue microarray comprising 30 paired LUAD cases. We found that MICAL-L2 was highly expressed in LUAD tissues when compared with that in matched paracancerous tissues (*P* < 0.001). LUAD tissues also exhibited strong MICAL-L2 staining in the nuclei of most tumor cells and the cytoplasm of some tumor cells (Figures 1A,B). c-Myc has been implicated in cancer progression and growth. The expression pattern of c-Myc was similar to that of MICAL-L2 (Figure 1C). Analysis of The Cancer Genome Atlas (TCGA) lung cancer dataset indicated that the expression of MICAL-L2 was significantly higher in cancer samples than in paracancerous tissues (Figure 1D). Next, we analyzed the expression of MICAL-L2 in a set of 4 NSCLC cell lines by immunoblotting, and found that MICAL-L2 expression was higher in most NSCLC cell lines compared with the non-malignant bronchial epithelial cell line BEAS-2B and the normal human fibroblast cell line MRC5 (Figures 1E,F). Overall, the clinical data indicated that MICAL-L2 expression was upregulated in lung cancer.

### MICAL-L2 Deficiency Downregulated c-Myc Expression in NSCLC Cells

**Figure 2:**
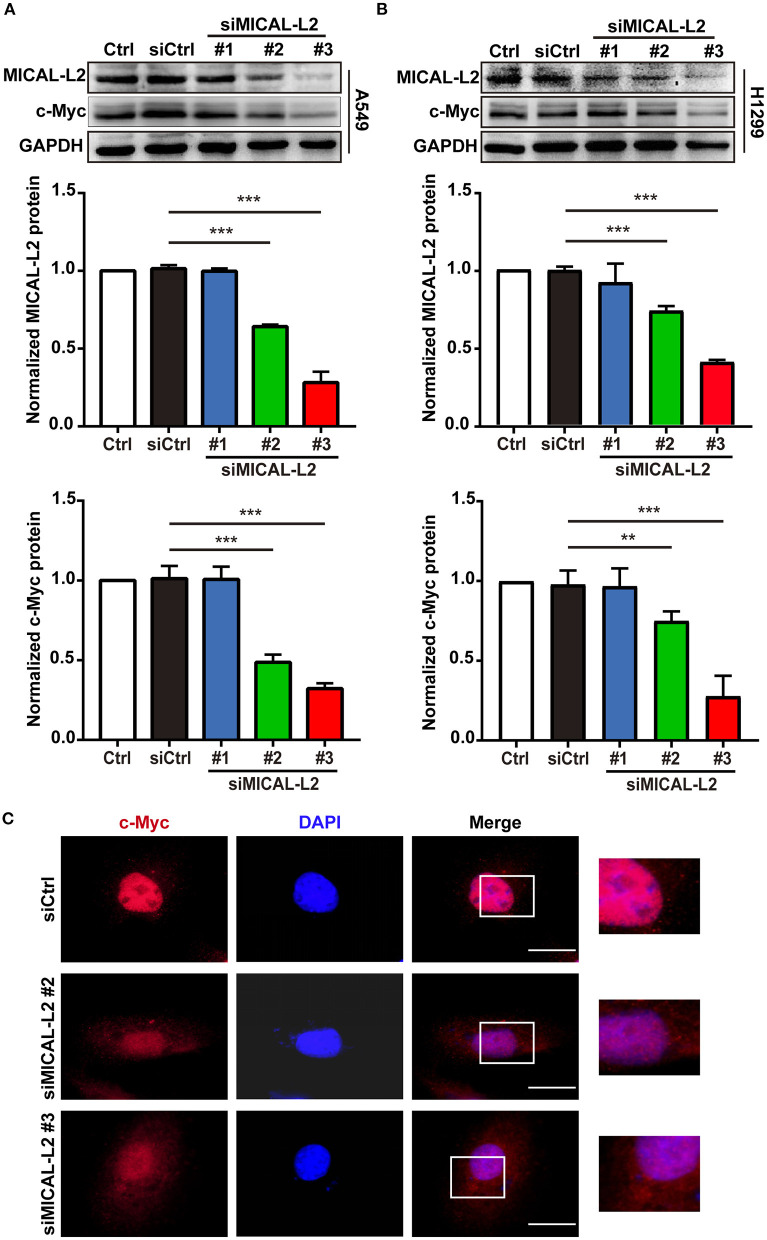
The effects of MICAL-L2 knockdown on c-Myc expression in non-small cell lung carcinoma (NSCLC) cells. **(A)** Total protein extracts from A549 cells treated with small interfering RNAs targeting MICAL-L2 (siMICAL-L2) for 48 h were assessed for MICAL-L2 and c-Myc expression. ***P < 0.001 relative to cells expressing control siRNA. **(B)** Blots showing the protein expression of MICAL-L2 and c-Myc in lysates from H1299 cells transfected with siMICAL-L2. **P < 0.01, ***P < 0.001 relative to cells expressing control siRNA. Data in **(A)** and **(B)** are presented as means ± SD of 3 determinations. **(C)** Representative immunofluorescence images of c-Myc staining in A549 cells transfected with siMICAL-L2. Scale bar, 5 μm.

We subsequently explored whether MICAL-L2 plays a key role in c-Myc expression in NSCLC cells. We first knocked down MICAL-L2 expression in A549 cells using siRNA and determined the knockdown efficiency by Western blotting. As shown in Figure 2A, both siMICAL-L2 #2 and #3 significantly downregulated the expression of MICAL-L2. Moreover, siMICAL-L2 also suppressed the expression of c-Myc in these cells, and a similar result was found for c-Myc expression in MICAL-L2-depleted H1299 cells (Figure 2B). Immunofluorescence staining showed that, in A549 cells transfected with siMICAL-L2 (#2 and #3), c-Myc was mainly detected in the nucleus and its fluorescence was decreased in MICAL-L2-knockdown A549 cells when compared with that in control cells (Figure 2C and Supplementary Figure 1A). No significant translocation of c-Myc from the nucleus to the cytoplasm was detected in these cells (Supplementary Figure 2).

### MICAL-L2 Positively Regulates NSCLC Cell Proliferation

**Figure 3:**
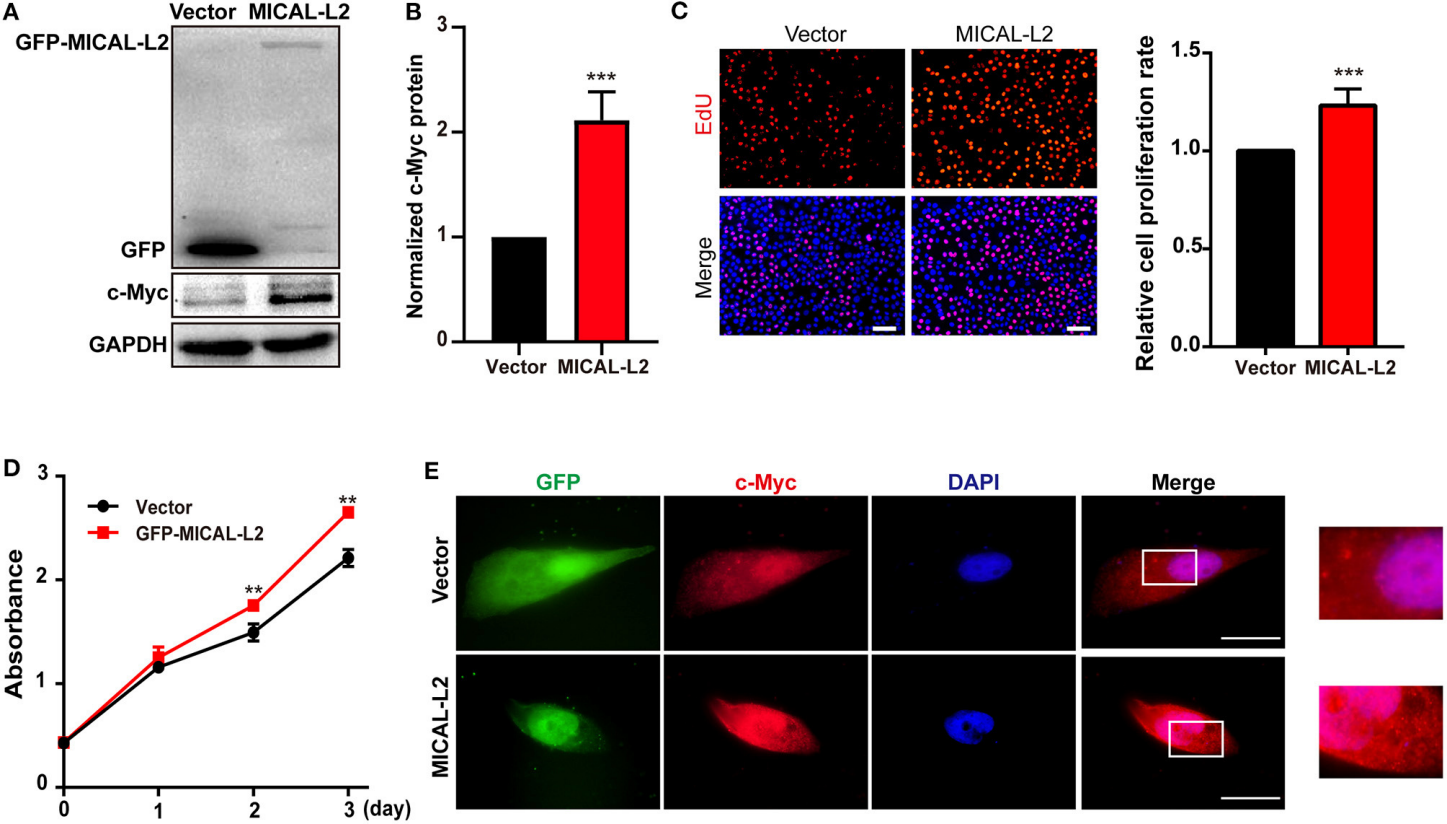
MICAL-L2 overexpression enhanced the proliferative ability of human non-small cell lung carcinoma (NSCLC) cells. **(A,B)** Total protein extracts from PC9 cells transfected with MICAL-L2 expression plasmids for 48 h were assessed for c-Myc expression. Data are presented as means ± SD of 3 determinations. ***P < 0.001 relative to control cells. **(C)** EdU staining in MICAL-L2-overexpressing PC9 cells. Data are presented as means ± SD of 5 independent determinations. **(D)** The viability of PC9 cells transfected with empty vector or MICAL-L2 expression plasmids was measured by CCK-8 assay. Data are presented as means ± SD of 10 determinations. **(E)** Representative immunofluorescence images of c-Myc staining in PC9 cells transfected with MICAL-L2 expression plasmids. Scale bar, 5 μm.**P < 0.01, ***P < 0.001 relative to control cells.

**Figure 4:**
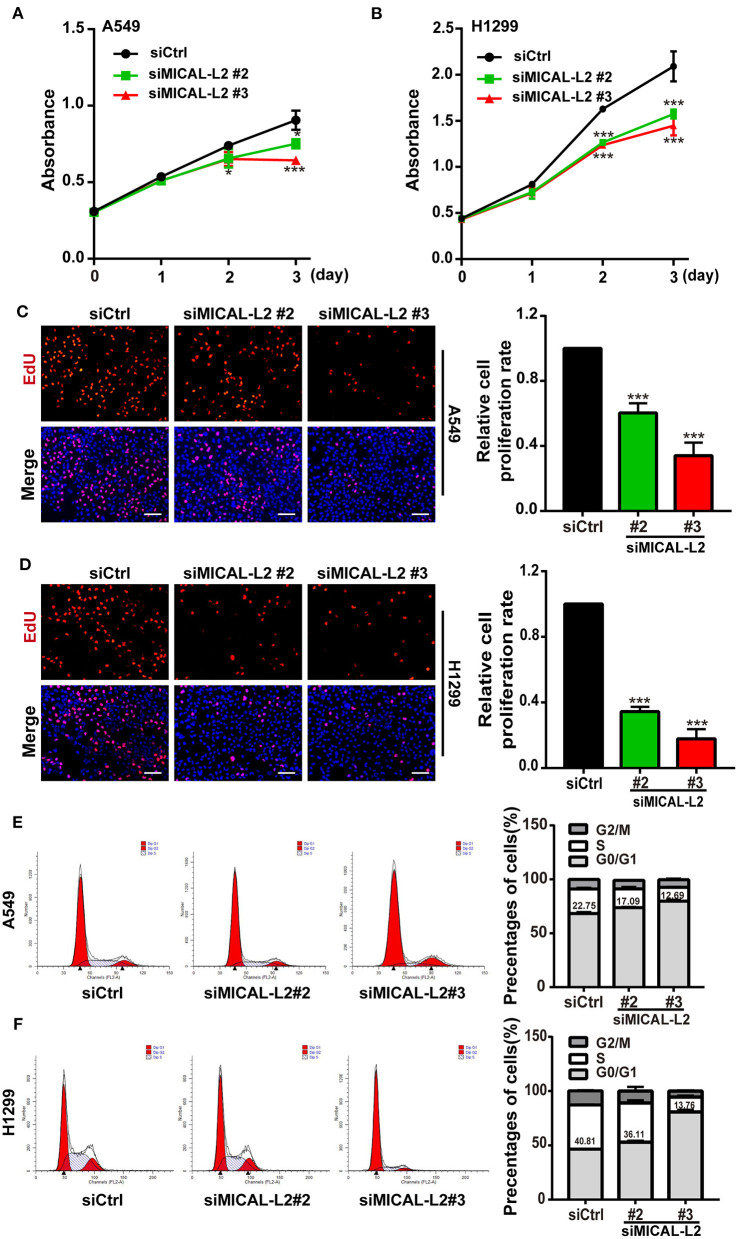
MICAL-L2 knockdown decreased the growth of non-small cell lung carcinoma (NSCLC) cells and induced S-phase cell cycle arrest. **(A,B)** CCK-8 assays for the viability of MICAL-L2-depleted A549 cells (left) and H1299 cells (right). **(C,D)** EdU staining showing the effect of MICAL-L2 depletion on the proliferative ability of A549 cells **(C)** and H1299 cells **(D)**. **(E,F)** A549 cells **(E)** and H1299 cells **(F)** underwent cell cycle distribution analysis by flow cytometry. Cell cycle distribution data are shown in histograms. *P < 0.05, ***P < 0.001 relative to control cells.

c-Myc expression is known to enhance the proliferation rates of various types of cells, including lung cancer cells. We noticed that after ectopic expression of MICAL-L2, both c-Myc expression (Figures 3A,B) and the proliferative potential of PC9 cells were increased (Figure 3C). EdU and CCK-8 incorporation assays also indicated that the proliferative ability of MICAL-L2-overexpressing PC9 cells was increased compared with that in the control group (Figures 3C,D). Immunofluorescence analysis further showed that c-Myc expression was markedly increased in PC9 cells overexpressing MICAL-L2 (Figure 3E and Supplementary Figure 1B). In contrast, pretreatment with MICAL-L2 siRNA significantly decreased the proliferative capacity of A549 and H1299 cells (Figures 4A–D). Flow cytometric analysis showed that knocking down MICAL-L2 in A549 and H1299 cells increased the number of cells in the G0/G1-phase of the cell cycle and decreased that in the S-phase. This implied that MICAL-L2 positively regulates the proliferative phase of NSCLC cells, particularly the G1 to S-phase transition of the cell cycle (Figures 4E,F).

### MICAL-L2 Maintains c-Myc Levels by Attenuating c-Myc Degradation

**Figure 5:**
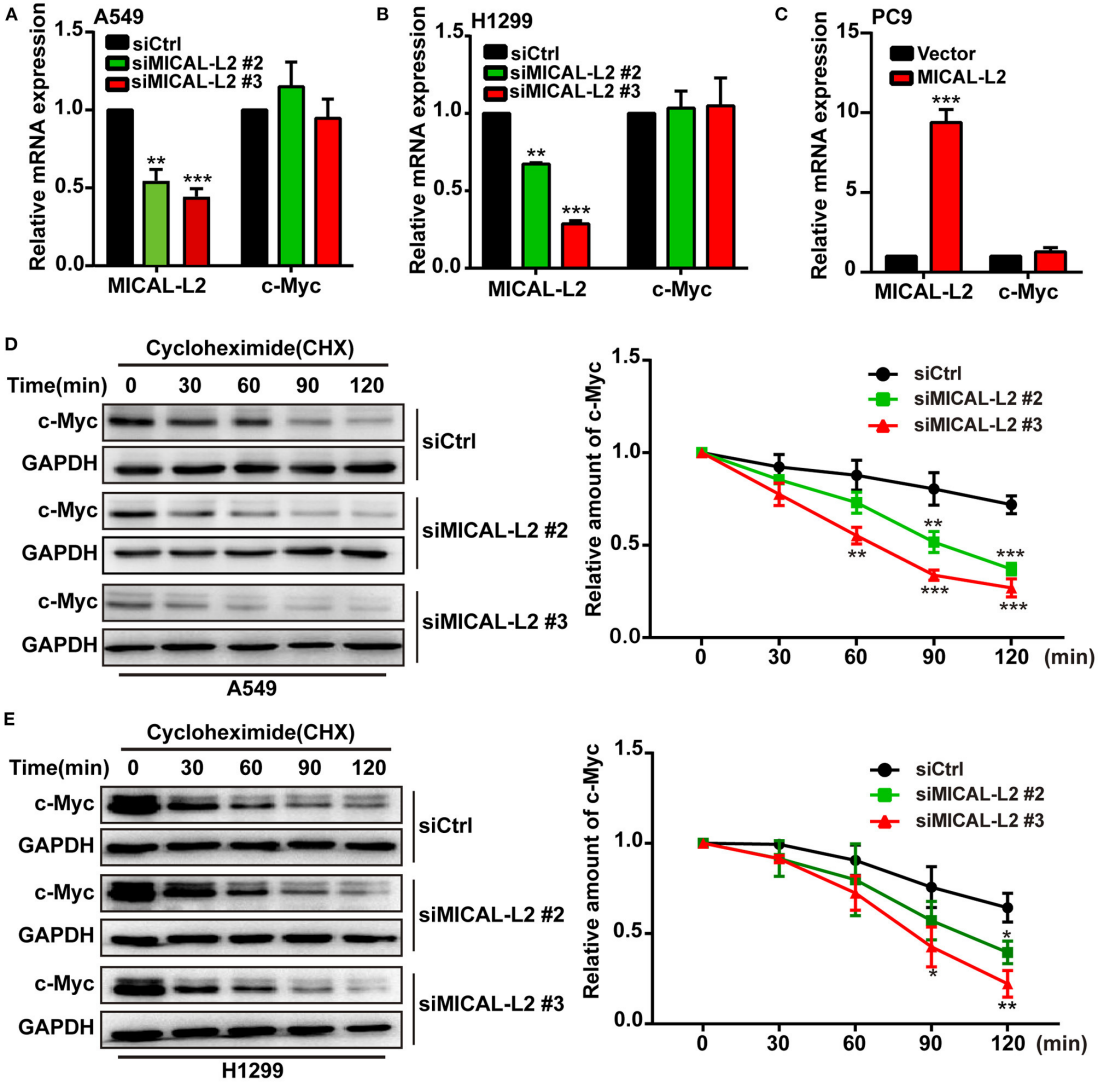
MICAL-L2 reduced c-Myc degradation. **(A,B)** The mRNA levels of MICAL-L2 and c-Myc were determined by RT-qPCR in A549 cells (left) and H1299 cells (right) transfected with small interfering (si) RNAs targeting MICAL-L2 (siMICAL-L2) and **(C)** PC9 cells transfected with MICAL-L2 expression plasmids. **(D,E)** The protein levels of c-Myc were examined in A549 and H1299 cells transfected with control siRNA or siMICAL-L2 and treated with cycloheximide (CHX, 10 μg/mL) for the indicated times. *P < 0.05, **P < 0.01, ***P < 0.001 relative to cells expressing control siRNA.

To investigate the mechanism involved in the MICAL-L2-dependent regulation of c-Myc expression, the mRNA levels of c-Myc in MICAL-L2-knockdown or overexpressing A549, H1299, and PC9 cells were analyzed by RT-qPCR. Despite a marked increase or decrease in MICAL-L2 expression, c-Myc mRNA levels were not significantly altered (Figures 5A–C). This suggested that MICAL-L2 might positively regulate c-Myc expression through different mechanisms, such as by disrupting its degradation. Consequently, we then knocked down MICAL-L2 expression in both A549 and H1299 cells by siRNA and examined c-Myc protein expression after treating the cells with CHX, a translation blocker. The results showed that knockdown of MICAL-L2 promoted c-Myc degradation relative to control cells (Figures 5D,E). The above results suggested that MICAL-L2 may help maintain c-Myc stability by reducing its degradation. The results further showed that MICAL-L2 depletion reduced the cyclin-D1 content, whereas ectopic c-Myc expression markedly upregulated cyclin-D1 levels in MICAL-L2-depleted cells (Supplementary Figure 3).

**Figure 6:**
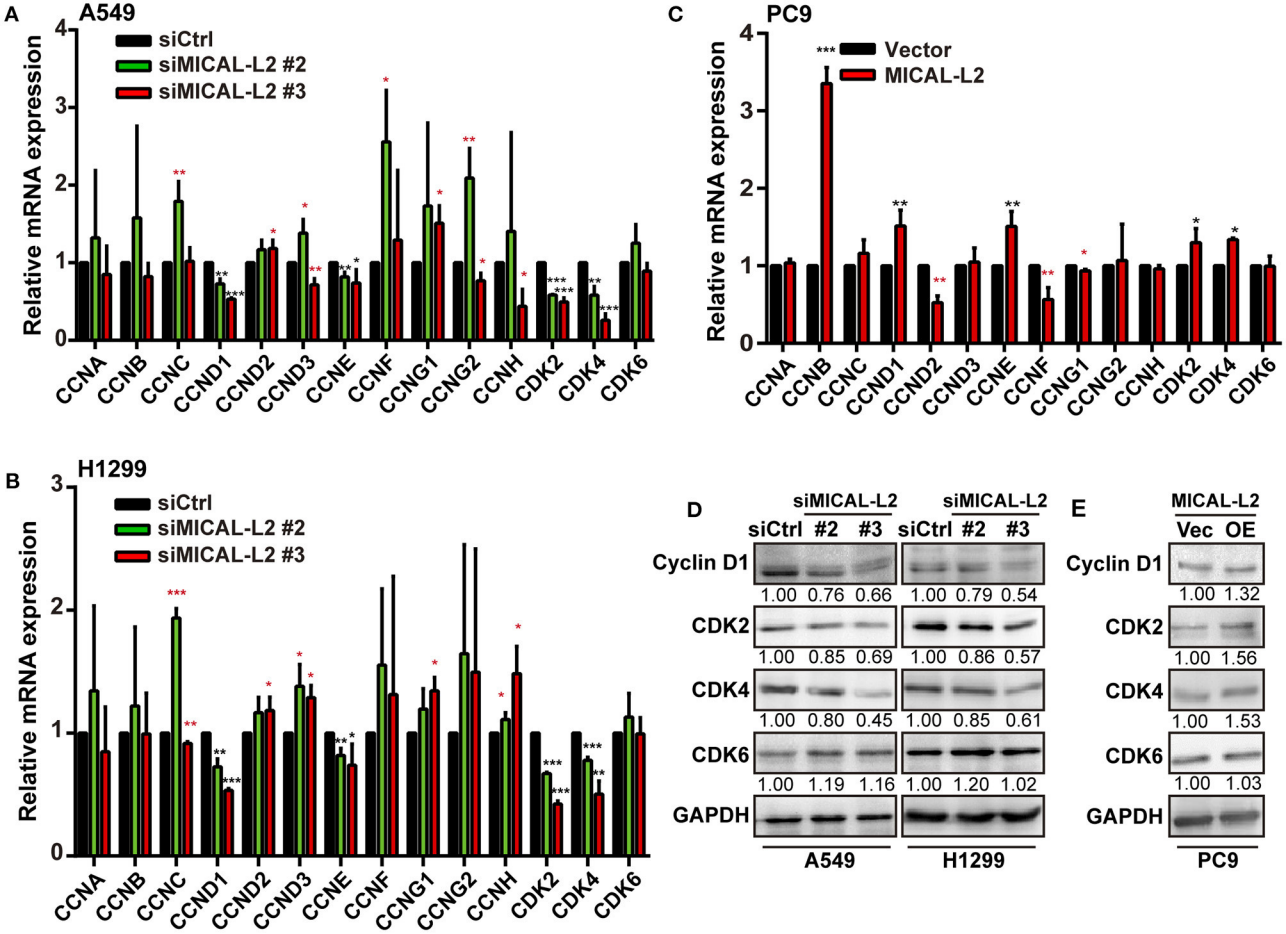
The effect of MICAL-L2 on cell cycle-related proteins. **(A,B)** RT-qPCR analysis of *CDK2, CDK4, CDK6*, and *CCNA-H* mRNA levels in A549 and H1299 cells transfected with control small interfering (si) RNA or siRNA targeting MICAL-2 (siMICAL-2). *P < 0.05, **P < 0.01, ***P < 0.001 relative to cells expressing control siRNA. **(C)** RT-qPCR analysis of *CDK2, CDK4, CDK6*, and *CCNA-H* mRNA levels in A549 cells transfected with empty vectors or MICAL-L2 expression plasmids. **(D,E)** Western blotting analysis of cyclin-D1, CDK2, CDK4, and CDK6 protein levels in MICAL-L2-depleted **(D)** or MICAL-L2-overexpressing **(E)** A549 cells. *P < 0.05, **P < 0.01, ***P < 0.001 relative to control cells.

To further confirm that the inhibitory effects on proliferation exerted by MICAL-L2 knockdown were mediated through a reduction in c-Myc protein levels, we then examined the expression of cell cycle-related genes in siMICAL-L2-transfected cells by qPCR. The results showed that the depletion of MICAL-L2 led to a significant decrease in the mRNA and protein levels of CCND1, CDK2, and CDK4 when compared with controls (Figures 6A,B,D). Meanwhile, as shown in Figures 6C,E, MICAL-L2 overexpression led to a marked increase in the mRNA and protein levels of CCND1, CDK2, and CDK4. In addition to their roles in regulating the G1 to S-phase transition of the cell cycle, these genes are also key c-Myc targets.

### MICAL-L2 Regulates c-Myc Stability via Suppressing Ubiquitin-Mediated c-Myc Degradation

**Figure 7:**
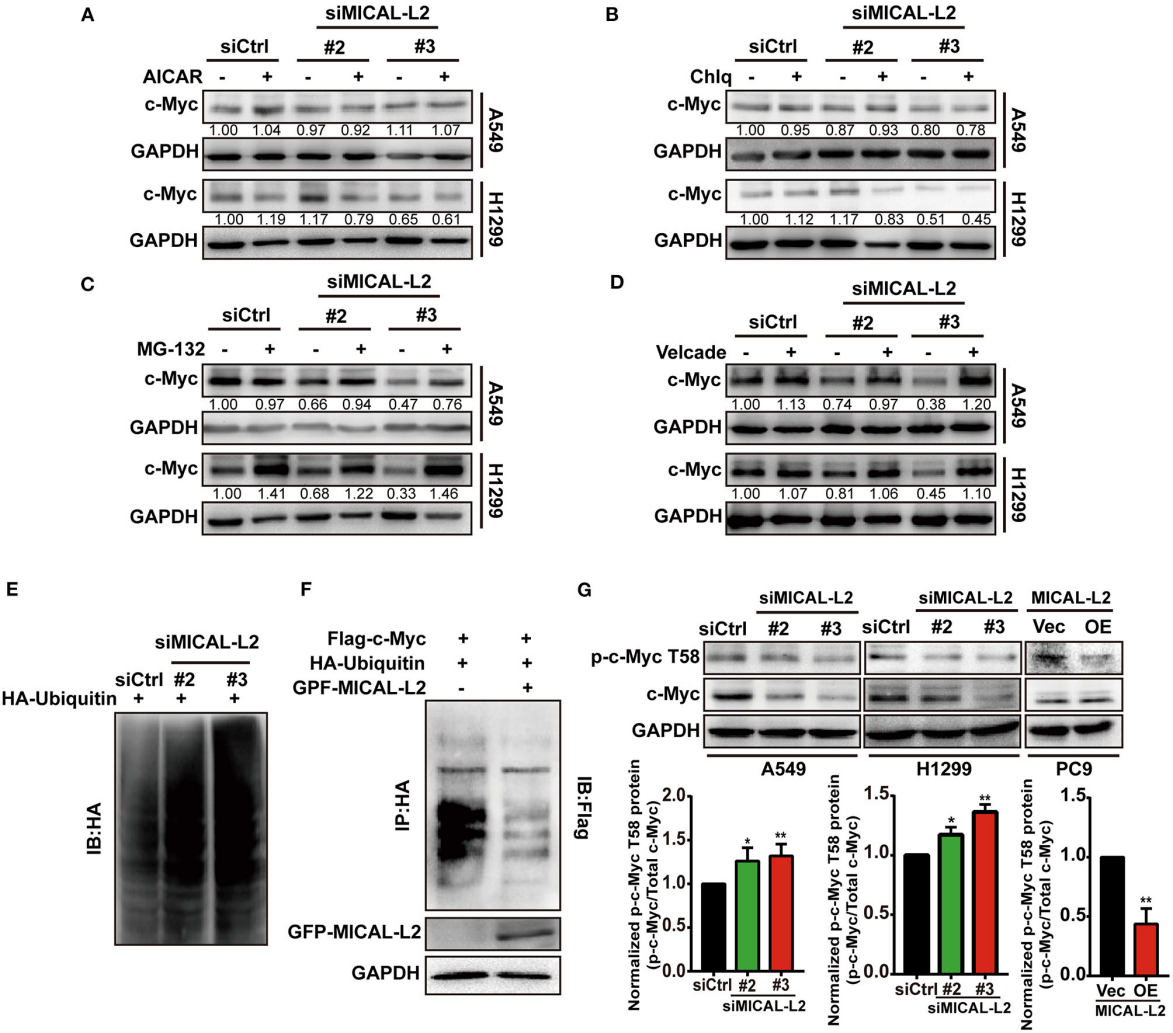
MICAL-L2 inhibited c-Myc ubiquitin-mediated degradation. **(A–D)** A549 and H1299 cells transfected with small interfering (si) RNA targeting MICAL-L2 (siMICAL-L2) were treated with AICAR (0.2 mM), chloroquine (10 μM), MG-132 (20 μM), and Velcade (10 μM) for 24 h. Total proteins were then extracted from the lysates and subjected to Western blotting to detect the expression of c-Myc. GAPDH served as the loading control. **(E)** H1299 cells were transfected with HA-ubiquitin and siMICAL-L2 and c-Myc polyubiquitination was detected by Western blotting using the indicated antibodies. **(F)** H1299 cells were co-transfected with HA-ubiquitin and GFP-MICAL-L2, Flag-c-Myc, or empty vector, following which c-Myc polyubiquitination was assayed. **(G)** A549 and H1299 cells were transfected with control siRNA or siMICAL-2. Total proteins were then extracted and analyzed for the expression of phosphorylated-c-Myc (T58) by Western blotting. PC9 cells were transfected with empty vector or MICAL-L2 expression plasmids. Total proteins were then extracted and analyzed for the expression of phosphorylated-c-Myc (T58) by Western blotting. Western blotting bands corresponding to phosphorylated-c-Myc/c-Myc were quantified and normalized against GAPDH. *P < 0.05, **P < 0.01 relative to control cells.

c-Myc degradation is known to be regulated via multiple pathways. To determine which pathway is involved in the MICAL-L2-mediated degradation of c-Myc, we treated cells with various inhibitors of known degradation pathways, namely, AICAR (macroautophagy inhibitor), chloroquine (lysosomal proteolysis inhibitor), MG-132 and Velcade (proteasome inhibitor). The results showed that only the proteasome inhibitor MG-132 and Velcade could block MICAL-L2 knockdown-induced c-Myc degradation (Figures 7A–D), suggesting that MICAL-L2 may inhibit c-Myc degradation via the proteasome pathway.

To further uncover the potential mechanism underlying c-Myc proteasomal degradation, we examined c-Myc ubiquitylation levels in MICAL-L2-depleted cells. As shown in Figure 7E, c-Myc polyubiquitylation levels were increased in H1299 cells transfected with siMICAL-L2. In contrast, c-Myc polyubiquitylation was significantly reduced following the ectopic expression of MICAL-L2 in H1299 cells (Figure 7F). Our data suggested that MICAL-L2 regulates c-Myc ubiquitylation in NSCLC cells.

c-Myc stability was reported to be highly correlated with low levels of threonine 58 (T58) phosphorylation. Additionally, pT58-c-Myc can be recognized by the E3 ubiquitin ligase complex SCF^Fbw7^, leading to c-Myc degradation through the proteasomal system (Yada et al., 2004). Consequently, we then evaluated the role of MICAL-L2 in modulating pT58-c-Myc expression using Western blotting. The pT58-c-Myc level was decreased in PC9 cells overexpressing MICAL-L2, but was increased in MICAL-L2-depleted A549 and H1299 cells (Figure 7G). The above results suggested that MICAL-L2 maintained c-Myc protein levels possibly through repressing polyubiquitylation-mediated c-Myc degradation.

### MICAL-L2 Interacts With c-Myc

**Figure 8:**
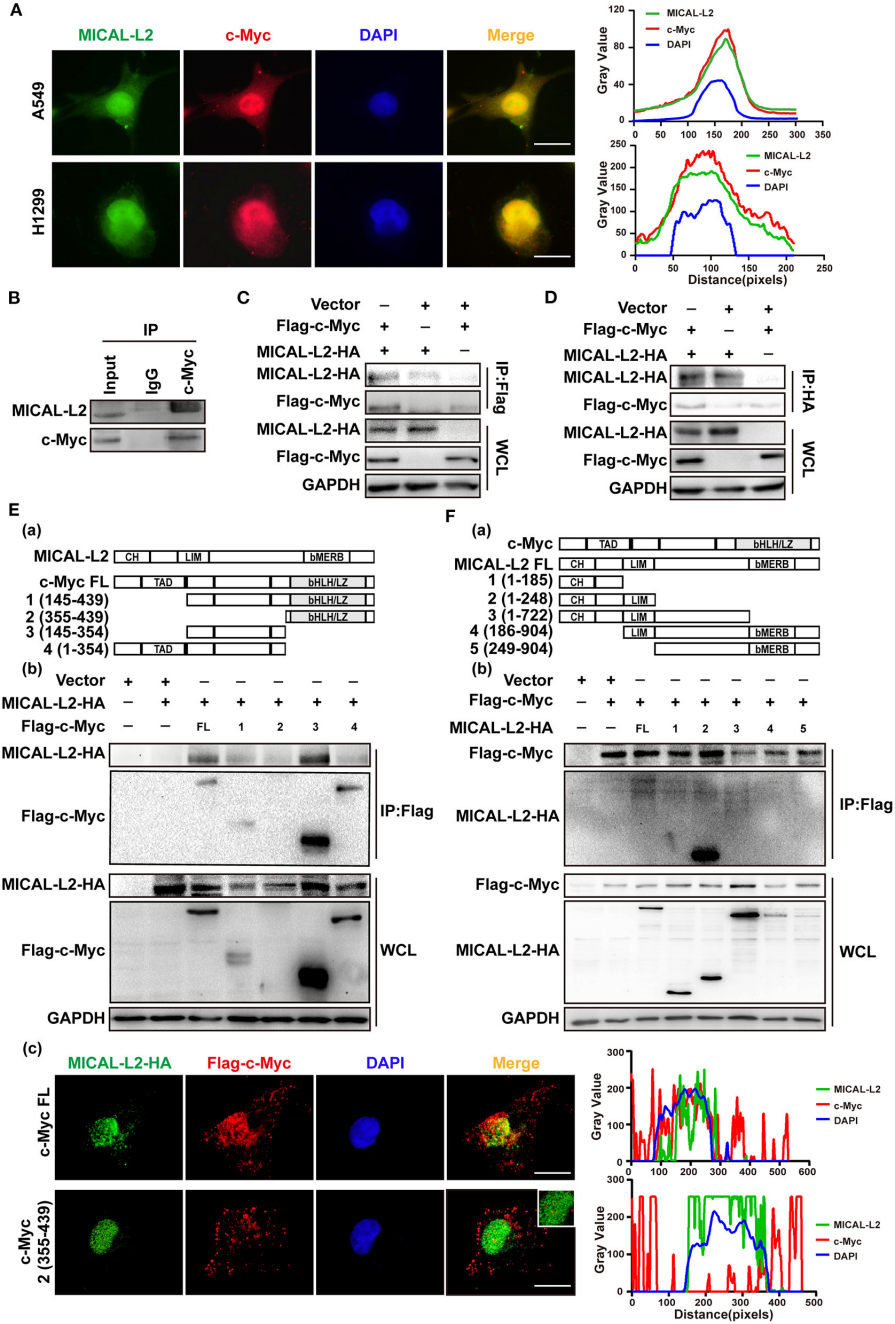
MICAL-L2 interacted with c-Myc. **(A)** Representative immunofluorescence images of MICAL-L2 (green), c-Myc (red), and nuclei (blue) staining in A549 and H1299 cells. **(B)** The binding of endogenous MICAL-L2 to c-Myc was detected in H1299 cells by co-immunoprecipitation assays. **(C,D)** Co-immunoprecipitation was performed with extracts from Cos-7 cells co-transfected with Flag-tagged c-Myc and HA-tagged MICAL-L2. **(E)** Schematic representation of the c-Myc domains (a). Cos-7 cells were co-transfected with HA-MICAL-L2 and a c-Myc mutant following which cell extracts were analyzed by Western blotting (b). Cos-7 cells were co-transfected with HA-MICAL-L2 and c-Myc mutant #2 following which cell extracts were analyzed using Immunofluorescence (c). **(F)** Schematic representation of the MICAL-L2 domains (a). Cos-7 cells were co-transfected with Flag-c-Myc and a MICAL-L2 mutant following which cell extracts were analyzed by Western blotting (b).

The above results indicated that MICAL-L2 regulates c-Myc expression at the posttranscriptional level. We then further explored whether MICAL-L2 and c-Myc colocalize. The results of the immunofluorescence assay indicated that they were located in the same part of the cell (Figure 8A). The interaction between endogenous MICAL-L2 and c-Myc was also confirmed by coimmunoprecipitation assays using H1299 cells (Figure 8B). Moreover, Flag-tagged c-Myc pulled down HA-MICAL-L2 after the co-transfection of the two plasmids into Cos-7 cells (Figure 8C), while HA-MICAL-L2 could also pull down Flag-tagged c-Myc after co-transfection (Figure 8D). These results suggested that MICAL-L2 directly binds to c-Myc.

We subsequently divided c-Myc and MICAL-L2 into fragments based on their known domains (Figures 8Ea,Fa) and expressed them individually in Cos-7 cells. The results indicated that the central region of c-Myc (amino acids [aa] 145–354) was crucial for its interaction with MICAL-L2 (Figure 8Eb), and the CH+LIM domain region of MICAL-L2 (aa 1–248) was required for its interaction with c-Myc (Figure 8Fb). We noticed that MICAL-L2 construct #3, which contains the CH+LIM domains, did not interact with c-Myc. The CC region of MICAL1 can bind to its LIM domain, thereby mediating its autoinhibition. Because construct #3 included regions other than the CH+LIM domains, it was possible that these other regions might inhibit the function of the CH+LIM domain region and prevent its binding to c-Myc. Notably, construct #2 of c-Myc (aa 355–439) was a small-sized fragment that could not be detected by Western blotting. However, as shown in Figure 8Ec, this construct was successfully expressed in cells. immunofluorescence analysis indicated that construct #2 of c-Myc and MICAL-L2 did not colocalize.

**Figure 9:**
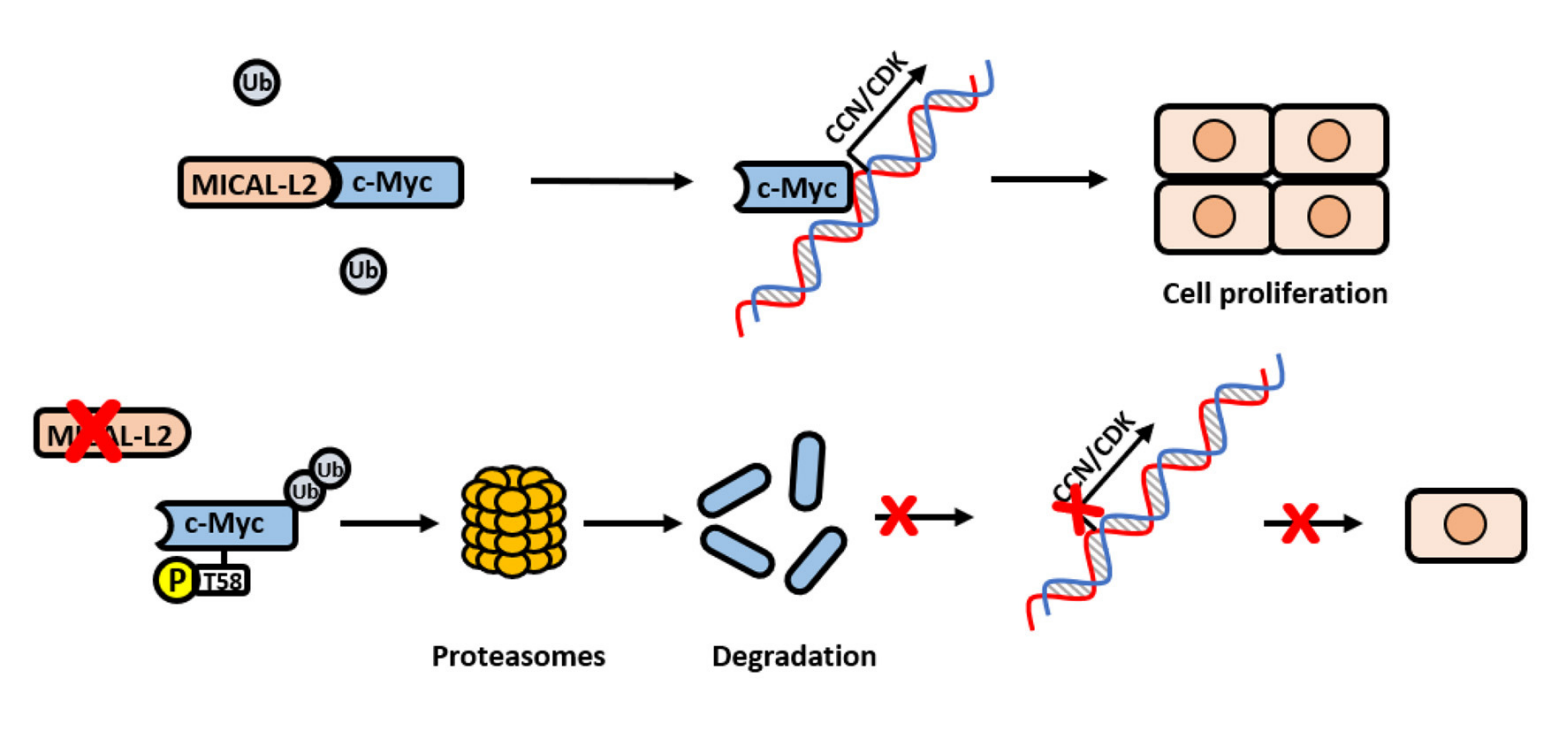
Schematic model for how MICAL-L2 regulates c-Myc expression and c-Myc-mediated cell proliferation. In brief, MICAL-L2 was identified as a novel key molecule implicated in non-small cell lung carcinoma (NSCLC) cell proliferation. MICAL-L2 binds to c-Myc, thereby suppressing c-Myc ubiquitination and degradation, which increases the expression of c-Myc target genes (including cell cycle-related genes) to promote NSCLC cell proliferation.

Overall, our clinical and *in vitro* data supported that MICAL-L2 may promote NSCLC cell proliferation via the c-Myc pathway (Figure 9).

## Discussion

In this paper, MICAL-L2, as a CasL interacting protein, preferentially binds to c-Myc. Notably, our report is the first to reveal such interaction in detail at the molecular level by performing binding domain analysis between MICAL-L2 and c-Myc. By interaction with c-Myc, MICAL-L2 is not only involved in this protein stability, but also participated in the subcellular c-Myc signaling pathway of cell proliferation. Furthermore, analysis of clinic specimens revealed increased expression of MICAL-L2 in lung adenocarcinoma tissues. Taken together, our results show that MICAL-L2 is a novel oncoprotein in NSCLC pathogenesis. We also reveal a characteristic of MICAL-L2 which could function as a regulator for c-Myc-mediated NSCLC cell proliferation.

Evidence from an increasing number of studies has indicated that the MICAL protein family, which includes MICAL1/2/3 and MICAL-L1/2, are important regulators of actin cytoskeleton dynamics and membrane trafficking (Terai et al., 2006; Giridharan et al., 2012; Fremont et al., 2017). We have previously reported that MICAL2 is involved in the regulation of breast cancer cell migration through the suppression of EGFR degradation (Wang et al., 2018). MICAL-L2, which lacks the MO domain and owns CC region compared with MICAL2 (Cai et al., 2018), is also a key member of the MICAL family and is frequently reported to serve as a cargo recognition protein (Sun et al., 2016). MICAL-L2 is well-known as an effector protein of Rab13. Accumulating evidence has indicated that the Rab13/MICAL-L2 complex is a critical regulator of epithelial junctional assembly, cytoskeletal reorganization, and neurite outgrowth (Nakatsuji et al., 2008; Sakane et al., 2010, 2012). MICAL-L2 is also involved in glucose transporter 4 trafficking as an effector of insulin-activated Rab13 (Sun et al., 2016). It is increasingly recognized that, although Rab is the main MICAL-L2 interaction partner, other proteins can also bind MICAL-L2. Of note, although proteomic profiling has shown that MICAL-L2 can interact with c-Myc (Agrawal et al., 2010), its oncogenic impact has never been explored. In MTD-1A epithelial cells, MICAL-L2 was detected both at cell-cell tight junctions and in the cytoplasm (Terai et al., 2006), while in L6 myoblasts GFP-MICAL-L2 was shown to be distributed along cytoplasmic filament-like structures. In this study, staining for MICAL-L2 in the NSCLC cell lines A549 and H1299 was primarily localized to the cytoplasm and nucleus. The different intracellular localization of MICAL-L2 may have been due to the different cell types used. The major finding of the present study was the identification of the link between MICAL-L2 and c-Myc. Consistent with the role of MICAL2 in promoting p53 ubiquitination during colorectal cancer development (Lu et al., 2018), our data indicated that MICAL-L2 knockdown shortened the half-life of c-Myc and decreased c-Myc protein levels, suggesting that MICAL-L2 may promote NSCLC cell proliferation through maintaining c-Myc function.

In the present study, we found that MICAL-L2 positively regulated c-Myc at the protein level, but not at the mRNA level. MICAL-L2 knockdown increased c-Myc polyubiquitination, whereas MICAL-L2 overexpression elicited the opposite effect. These results suggested that MICAL-L2 might modulate c-Myc expression through the ubiquitin-proteasome system rather than through a transcription-dependent mechanism. We also found that MICAL-L2 blocked c-Myc polyubiquitination and degradation, presumably by inhibiting c-Myc phosphorylation at T58. As p-T58 can recruit SCF^Fbw7^, a component of the SCF E3 ubiquitin ligase complex, to c-Myc (Welcker et al., 2004; Yada et al., 2004), T58 dephosphorylation may play an important role in preventing c-Myc polyubiquitination and its subsequent degradation. Moreover, we identified the domains in MICAL2 and c-Myc that are likely to be involved in their interaction. c-Myc is a multifaceted transcription factor that has been suggested to regulate the expression of up to 15% of all human genes. Recent evidence indicates that c-Myc regulation may be context-dependent; for instance, some transcription factors or cofactors were reported to determine the outcome of c-Myc-mediated transcription (Wu et al., 2017). Here, we found that the interaction between MICAL-L2 and c-Myc was related to cell proliferation, as MICAL-L2 could regulate the expression of c-Myc target genes (Dang, 1999), including *CCND1, CCNE, CDK2*, and *CDK4*, as well as the protein expression of CCND1, CDK2, and CDK4. Together, these results further support that MICAL-L2-mediated c-Myc stability is critical for NSCLC cell proliferation.

Because cell proliferation is a complex process involving multiple cellular pathways, MICAL-L2 could potentially act through several different signaling pathways to achieve the same result. Mechanistically, MICAL-L2 was shown to be significantly upregulated in ovarian cancer tissues and to activate the Wnt/beta-catenin signaling pathway (Zhu et al., 2015). Here, we have described a novel role for MICAL-L2 in preventing c-Myc ubiquitination and degradation in the nucleus. This results in the increased expression of c-Myc target genes, including cell cycle-related genes, as well as the promotion of NSCLC cell proliferation, which may finally lead to NSCLC development. Further *in vivo* studies are needed to elucidate how MICAL-L2 regulates NSCLC growth and whether MICAL-L2 may be a potential therapeutic target for the treatment of this cancer.

## Data Availability Statement

The raw data supporting the conclusions of this article will be made available by the authors, without undue reservation.

## Ethics Statement

The studies involving human participants were reviewed and approved by Ethics Committee of Nanjing Medical University. The patients/participants provided their written informed consent to participate in this study.

## Author Contributions

JD and PM designed the study. PM, LZ, and YW performed the experiments. PM, CQ, YS, MB, YZ, and YM performed the statistical analysis. JD, PM, XZ, and MY drafted the manuscript. JD supervised the experimental work. All authors read and approved the final manuscript.

## Conflict of Interest

The authors declare that the research was conducted in the absence of any commercial or financial relationships that could be construed as a potential conflict of interest.

## Supplementary Material

Supplementary Figure 1 The quantification of the fluorescence intensity of c-Myc in **(A)** A549 cells transfected with siMICAL-L2; and **(B)** PC9 cells transfected with MICAL-L2 plasmids.

Supplementary Figure 2 Knocking down MICAL-L2 suppressed c-Myc expression in the nucleus. A549 and H1299 cells transfected with siMICAL-L2 were lysed and c-Myc levels in the cytoplasm and nucleus were determined by Western blotting.

Supplementary Figure 3 c-My reversed the effect of MICAL-L2 siRNA on cyclin-D1 levels. A549 and H1299 cells were transfected with MICAL-L2 expression plasmids and siRNA as shown and cyclinD1 protein levels were determined by Western blotting.
