# Supplementary figures and images for: MICAL-L2 Is Essential for c-Myc Deubiquitination and Stability in Non-small Cell Lung Cancer Cells
Source: Front Cell Dev Biol. 2021 Jan 14;8:575903. doi: 10.3389/fcell.2020.575903 (PMC7841116; doi:10.3389/fcell.2020.575903)

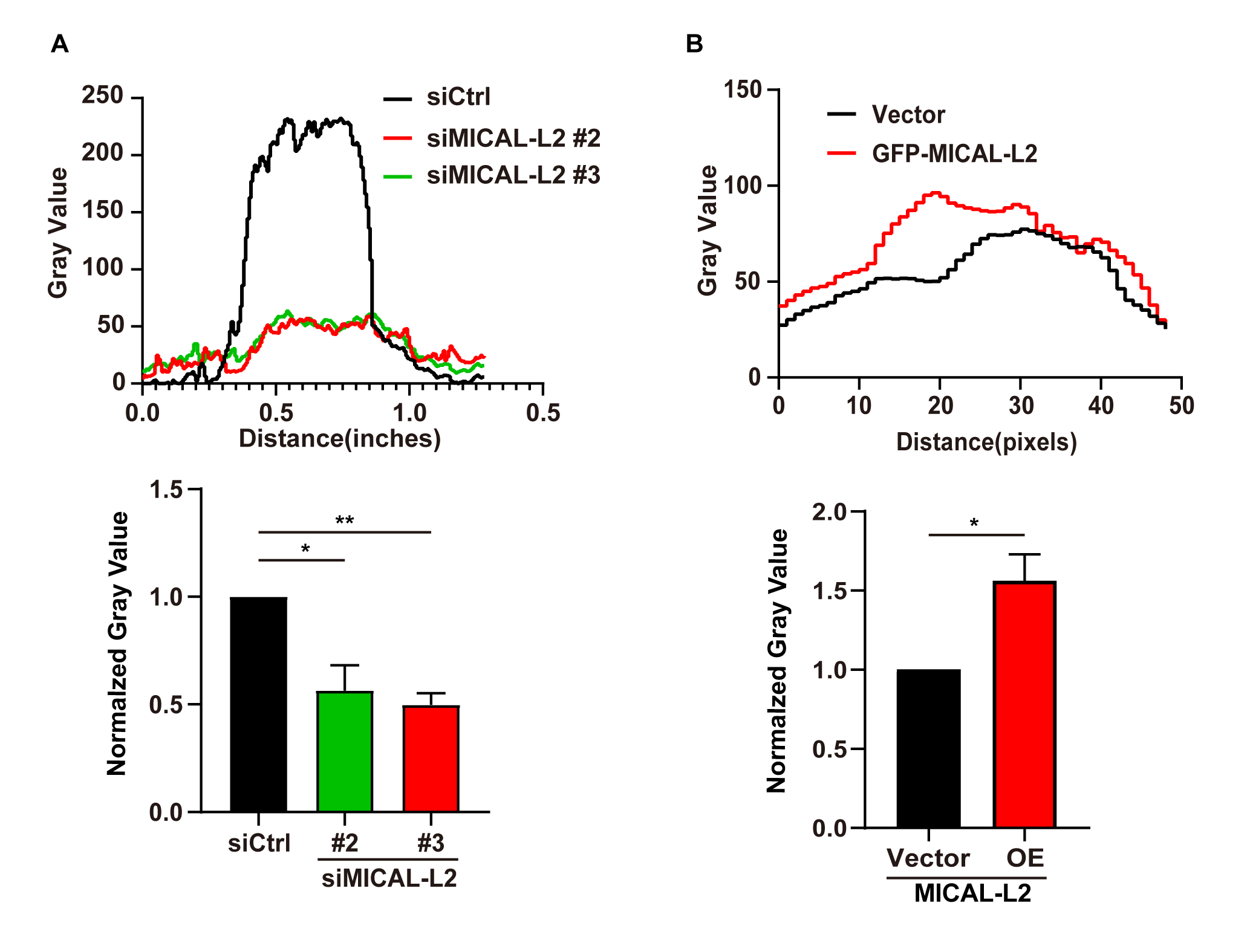

Supplement: Supplementary Figure 1 — The quantification of the fluorescence intensity of c-Myc in (A) A549 cells transfected with siMICAL-L2; and (B) PC9 cells transfected with MICAL-L2 plasmids. [file Image_1.TIF]

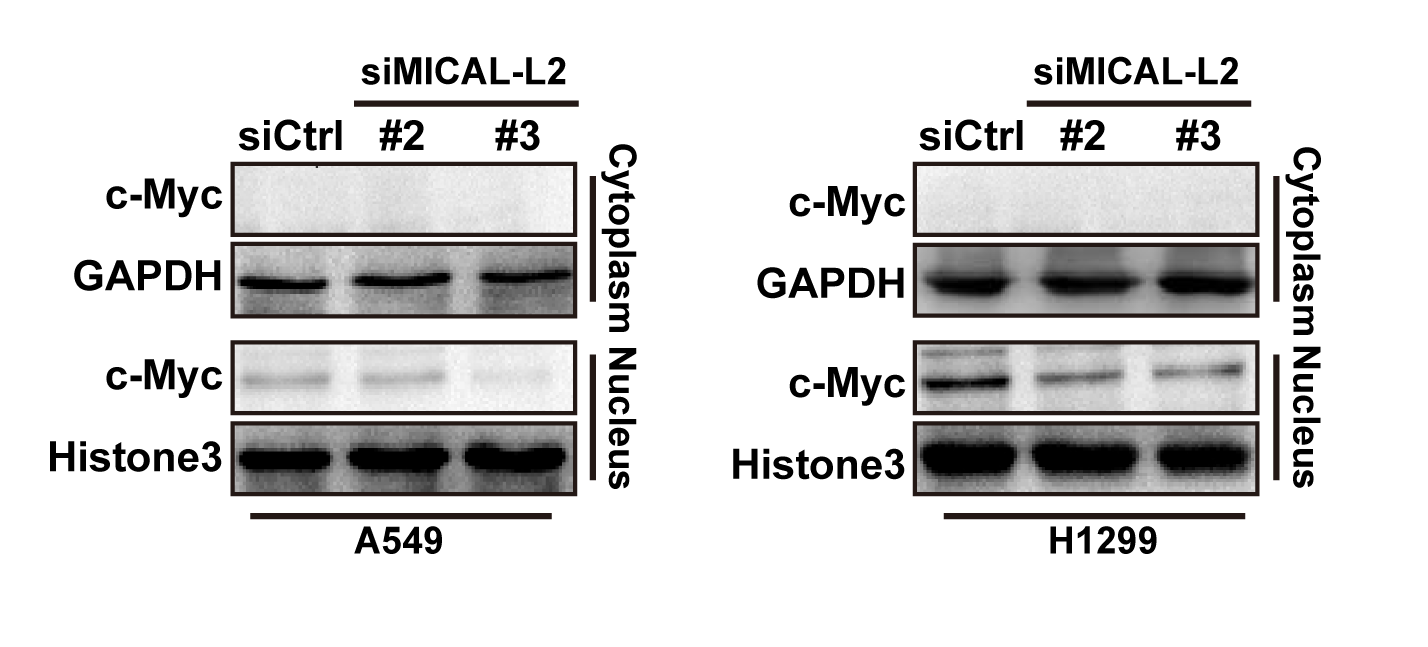

Supplement: Supplementary Figure 2 — Knocking down MICAL-L2 suppressed c-Myc expression in the nucleus. A549 and H1299 cells transfected with siMICAL-L2 were lysed and c-Myc levels in the cytoplasm and nucleus were determined by Western blotting. [file Image_2.TIF]

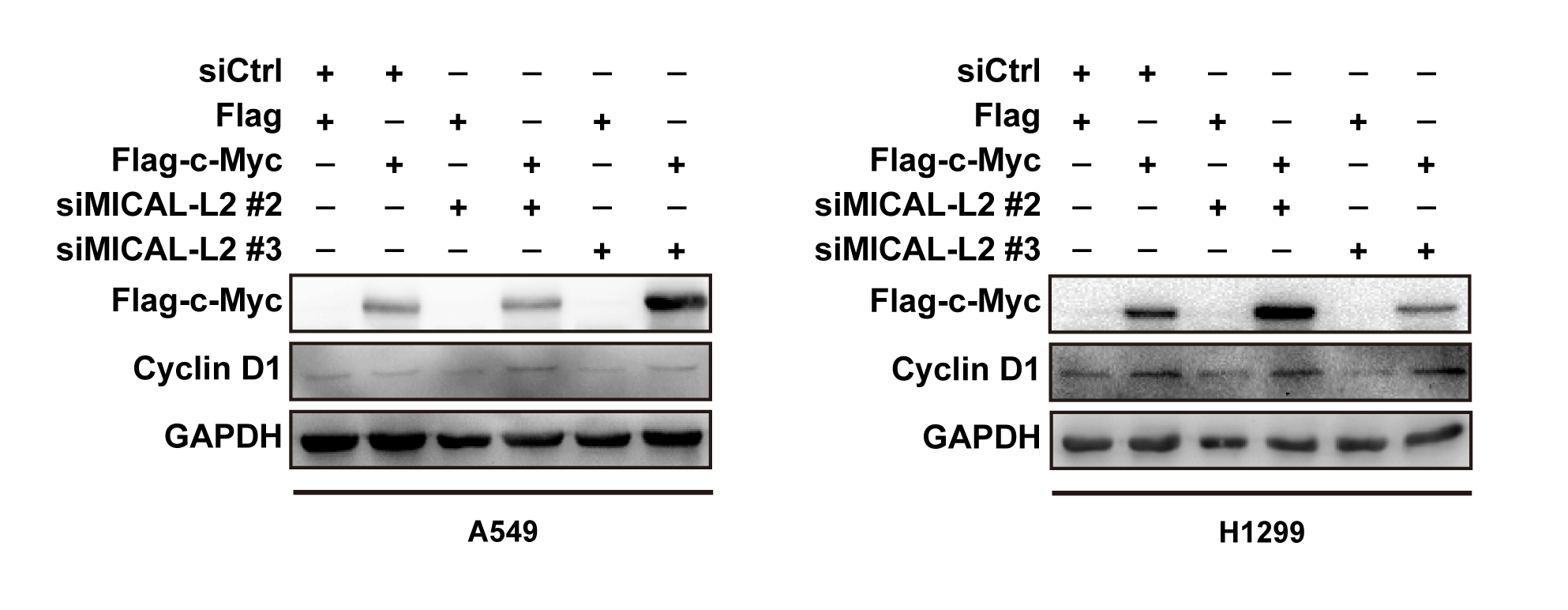

Supplement: Supplementary Figure 3 — c-My reversed the effect of MICAL-L2 siRNA on cyclin-D1 levels. A549 and H1299 cells were transfected with MICAL-L2 expression plasmids and siRNA as shown and cyclinD1 protein levels were determined by Western blotting. [file Image_3.TIF]
